# bta-miR-23a involves in adipogenesis of progenitor cells derived from fetal bovine skeletal muscle

**DOI:** 10.1038/srep43716

**Published:** 2017-03-03

**Authors:** Long Guan, Xin Hu, Li Liu, Yishen Xing, Zhengkui Zhou, Xingwei Liang, Qiyuan Yang, Shengyun Jin, Jinshan Bao, Huijiang Gao, Min Du, Junya Li, Lupei Zhang

**Affiliations:** 1 Institute of Animal Science, Chinese Academy of Agricultural Sciences, Beijing 100193, China; 2 Institute of Animal Husbandry, Heilongjiang Academy of Agricultural Sciences, Harbin 150086, China; 3 State Key Laboratory for Conservation and Utilization of Subtropical Agro-bioresources, Guangxi High Education Laboratory for Animal Reproduction and Biotechnology, Guangxi University, Guangxi 530004, China; 4 Department of Animal Sciences, Washington State University, Pullman, WA 99164, USA; 5 Animal Husbandry Station of Wulagai, Wulagai 026321, China

## Abstract

Intramuscular fat deposition or marbling is essential for high quality beef. The molecular mechanism of adipogenesis in skeletal muscle remains largely unknown. In this study, we isolated Platelet-derived growth factor receptor α (PDGFRα) positive progenitor cells from fetal bovine skeletal muscle and induced into adipocytes. Using miRNAome sequencing, we revealed that bta-miR-23a was an adipogenic miRNA mediating bovine adipogenesis in skeletal muscle. The expression of bta-miR-23a was down-regulated during differentiation of PDGFRα^+^ progenitor cells. Forced expression of bta-miR-23a mimics reduced lipid accumulation and inhibited the key adipogenic transcription factor peroxisome proliferative activated receptor gamma (*PPARγ*) and CCAAT/enhancer binding protein alpha (*C/EBPα*). Whereas down-regulation of bta-miR-23a by its inhibitors increased lipid accumulation and expression of *C/EBPα, PPARγ* and fatty acid-binding protein 4 (*FABP4*). Target prediction analysis revealed that *ZNF423* was a potential target of bta-miR-23a. Dual-luciferase reporter assay revealed that bta-miR-23a directly targeted the 3′-UTR of *ZNF423*. Together, our data showed that bta-miR-23a orchestrates early intramuscular adipogeneic commitment as an anti-adipogenic regulator which acts by targeting *ZNF423*.

Fetal stage is a crucial period for both skeletal muscle development and intramuscular preadipocyte formation^1^. During early skeletal muscle development, myogenic cells and intramuscular adipocyte are commonly derived from mesenchymal stem cells (MSCs) in embryonic mesoderm^2^,^3^. Parts of the MSCs firstly differentiate into either myogenic or non-myogenic lineages. The non-myogenic lineage progenitor cells have adipogenic and fibrogenic potential, as well as osteogenic and chondrogenic capacity^4^,^5^. Most of the non-myogenic progenitor cells subsequently differentiate into adipocytes and fibroblasts, while the others reside in the stromal-vascular fraction of mature skeletal muscle tissue without differentiation, forming the progenitor pool^6^. Platelet-derived growth factor receptor α (PDGFRα) is a specific surface marker of these non-myogenic progenitor cells^7^. Intramuscular adipogenesis from progenitor cells in prenatal stage provides the fat deposition sites for postnatal intramuscular fat (IMF) formation.

Adipogenesis is divided into commitment and differentiation. Several critical transcriptional factors (TFs) have been demonstrated to mediate adipogenesis commitment and differentiation. Using preadipocytes model, two TFs, CCAAT/enhancer binding protein alpha (C/EBPα) and peroxisome proliferative activated receptor gamma (PPARγ), were characterized as the crucial regulators of adipogenesis differentiation^8^,^9^. C/EBPα and PPARγ reinforce each other’s expression. Activation of these factors promote adipogenesis and increase lipid accumulation in cells^10^. PPARγ also plays the central role in bovine IMF adipogenesis^11^. C/EBPα and PPARγ expression in skeletal muscle are greater in the cattle breed with higher IMF deposition capacity^12^. Zinc finger protein 423 (ZNF423, also known as ZFP423) was identified as another important regulator and involved in both adipogenic commitment of progenitor cells and *PPARγ* activation^13^. Stromal vascular cells expressing ZNF423 have robust adipogenesis potential^14^. Overexpressing *ZNF423 in vitro* promoted the MEFs adipogenesis^15^. In farm animal, *ZNF423* could promote adipogenic differentiation in bovine skeletal muscle derived stromal vascular cells^16^.

Adipogenesis is also under post-translational regulation by microRNAs (miRNAs). miRNAs are small non-coding RNAs (nc-RNAs) with an average length of 22 nucleotides(nt). Mature miRNAs interact with target mRNAs at specific sites of 3′ untranslated regions (3′UTR) by base pairing. A single miRNA can have one to several hundred target mRNAs, meanwhile a single mRNA can have multiple miRNA binding sites in 3′UTR^17^. The binding sites of miRNAs often evolutionarily conserved, especially between bases 2–8 of their 5′ end (seed sequence)^17^. miRNAs bind to target mRNAs and induce their translational repression and/or deadenylation^18^,^19^. Being the key regulation factors, miRNAs play crucial roles in various biological process such as cell growth, differentiation and development. Many miRNAs are expressed in a tissue-specific^20^ and/or stage-specific manner^21^. A series of miRNAs including miR-27a/b, miR-143, miR-448, miR-130 and let-7, have been reported regulating adipogenesis in mice or human^22^,^23^,^24^,^25^,^26^. However, limited miRNAs have been reported to modulate adipogenesis in cattle^27^. Furthermore, although the miRNAs expression profiles in bovine subcutaneous fat and IMF have been characterized^28^,^29^,^30^, little research focused on the miRNAs expression characteristic of early IMF development in prenatal stage. Since the important role in fetal development and its profound impact on IMF deposition, the objective of this study is to characterize the miRNAome expression profile during adipogenesis and determine their role in adipogenic differentiation of bovine progenitor cells. Of note, the significance of this study is to help understand how miRNA regulated intramuscular development during fetal stage in bovine.

## Results

### PDGFRα^+^ progenitor cells isolation and adipogenic differentiation

**Figure 1:**
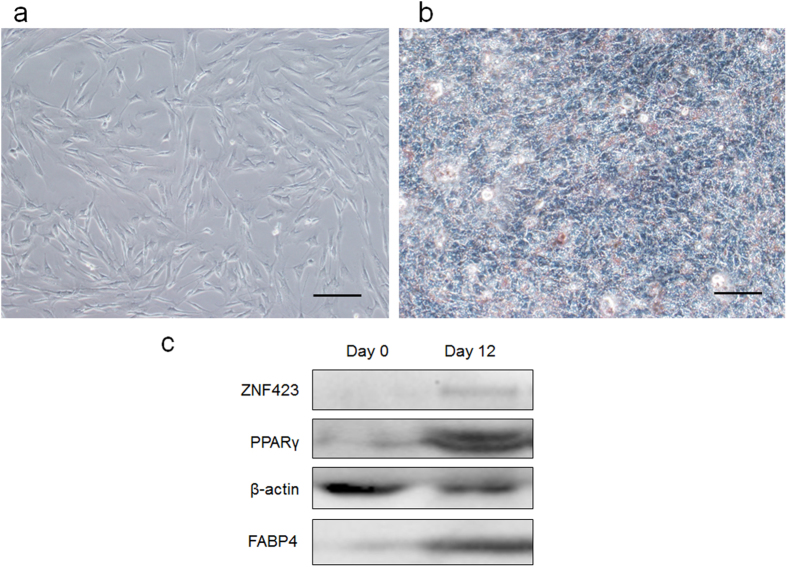
Adipogenic differentiation in PDGFRα^+^ progenitor cells. (**a**) PDGFRα^+^ progenitor cells isolated from bovine fetal skeletal muscle tissue. Scale bar, 100 μm. (**b**) Oil Red O staining of PDGFRα^+^ progenitor cells after adipogenic differentiation. Scale bar, 100 μm. (**c**) Immunoblot analysis of white adipogenic markers before or after differentiation, n = 3.

The primary cells isolated from the fetal skeletal muscle tissue adhered to the culture plates and began to elongate after 24 h. Approximately 3 days later, the cells exhibited a shuttle shape and grew to reach 70–80% confluence (Fig. 1a). The cell bodies appeared to have strong refraction.

After adipogenic differentiation, most of the cells changed from the shuttle shape into an oblate shape during the first 4 days. On the 6th day of induction, lipid microdroplets could be observed in some cells under microscope. The amount of lipid droplets increased in a time-dependent manner, and lipid microdroplets aggregated and fused to form larger droplets in this process (Fig. 1b). The results of Oil Red O staining indicated the presence of lipid in the cells at 4 days after induction. Immunoblotting data showed that adipocyte-specific markers ZNF423, PPARγ and fatty acid-binding protein (FABP4) significantly increased after differentiation (Fig. 1c).

### Small RNAs sequencing and identification of conserved miRNAs

**Figure 2:**
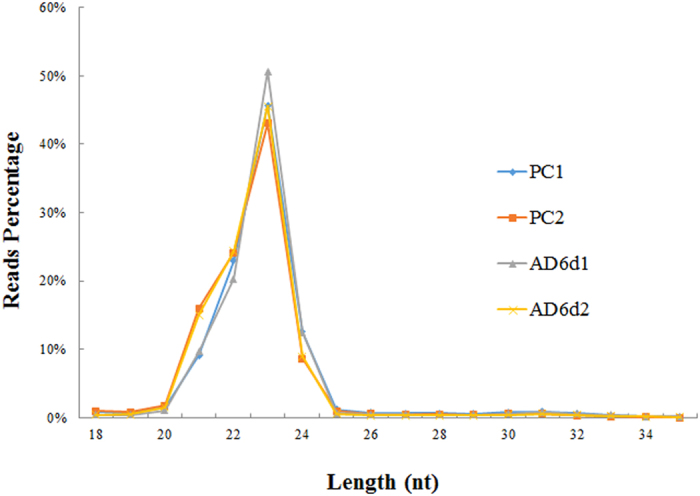
Length distribution of small RNA reads in PC and AD6d libraries.

To isolate the miRNAs functioning in adipogenesis, total RNA was extracted from progenitor cells (PC) and cells at day 6 of differentiation (AD6d). After generating the libraries, two datasets were obtained from PC and AD6d (PC1, 9232752 reads; PC2, 8451410 reads; AD6d1, 7672984 reads; AD6d2, 10241514 reads), respectively. Clean reads (about 97% of total reads) were obtained by trimming 3′ adapter sequence, and removing the reads containing ploy-N, with 5′ adapter contaminants, without 3′ adapter or the insert tag, containing poly A, T, G or C and low quality reads from raw data (Table 1). Then, the reads were classified by length as shown in Fig. 2. The most abundant size for miRNAs was 21–24 nucleotides. However, only miRNAs with a length range of 18–35 nt from clean reads were filtered for further downstream analyses. Subsequently, the small RNA tags were mapped to bovine reference sequence without mismatch using Bowtie. miRBase21 was used to identify conserved miRNAs as reference in mapped tags. The numbers of miRNA reads were normalized by Tags per million (TPM) values (TPM = (readCount*1,000,000)/libsize) to express miRNAs in PC and AD6d comparable in one table.

### Identification of differentially expressed miRNAs after adipogenic differentiation

**Figure 3:**
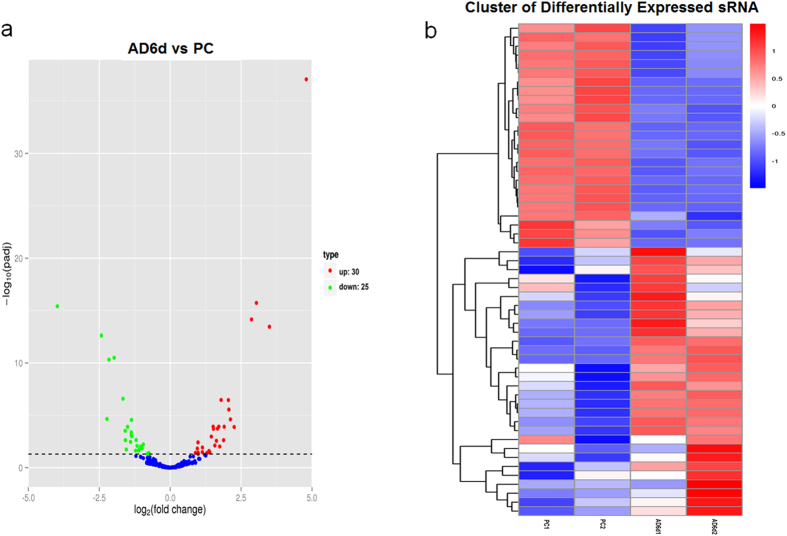
Differentially expressed miRNAs. (**a**) Differentially expressed miRNAs in volcano plot. The X-axis stands for the fold change of miRNAs. The Y-axis stands for significant difference of miRNA expression changes. Every miRNA are represented with the dots. The blue dots indicate miRNAs without significant difference; The red dots indicate up-regulated miRNAs; The green dots stand for down-regulated miRNAs. (**b**) Hierarchical clustering of miRNA expression. miRNA profiles from four libraries were clustered. Samples are in columns, and miRNAs are in rows. Cluster analysis based on log_10_ (TPM+ 1). The red indicates up-regulated miRNAs, and the blue indicates down-regulated miRNAs.

Differentially expressed conserved miRNAs between non-differentiated group (PC) and differentiating group (AD6d) were analyzed using DESeq R package. Based on the negative binomial distribution, volcano plot was generated to show the normalized miRNA expression levels (Fig. 3). We employed hierarchical cluster to analyze differentially expressed miRNAs of all samples (Fig. 3). Among the 55 differentially expressed miRNAs, 30 miRNAs were up-regulated and 25 miRNAs were down-regulated after adipogenic induction (Supplementary Table 1).

**Figure 4:**
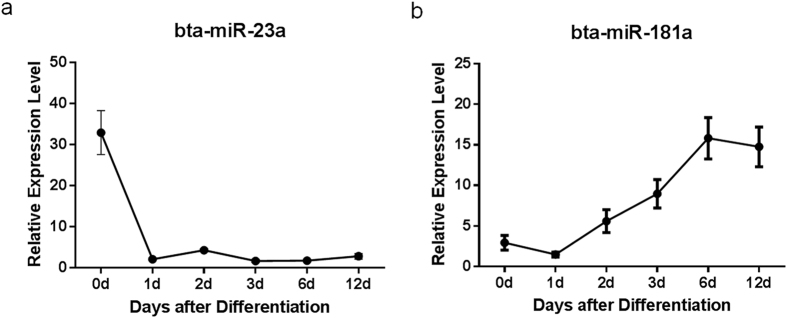
Expression of bta-miR-23a and bta-miR-181a during adipogenic differentiation. Quantitative RT-PCR assays of bta-miR-23a (**a**) and bta-miR-181a (**b**) expression in progenitor cells throughout differentiation. Data are representative of three independent experiments and given as means ± S.E.M. n = 3.

To validate the differentially expressed miRNAs, we selected bta-miR-181a, an up-regulated miRNA, and bta-miR-23a, a down-regulated miRNA to assay their expression levels during differentiation. Their expression during d0 to d12 of differentiation was quantified (Fig. 4). bta-miR-23a expression decreased substantially 1 day after induction and kept a relative low level in the following differentiation. In contrast, bta-miR-181a expression gradually increased from induction and kept at relative high level after day 6.

### miR-23a inhibits adipogenic differentiation of PDGFRα^+^ progenitor cells

**Figure 5:**
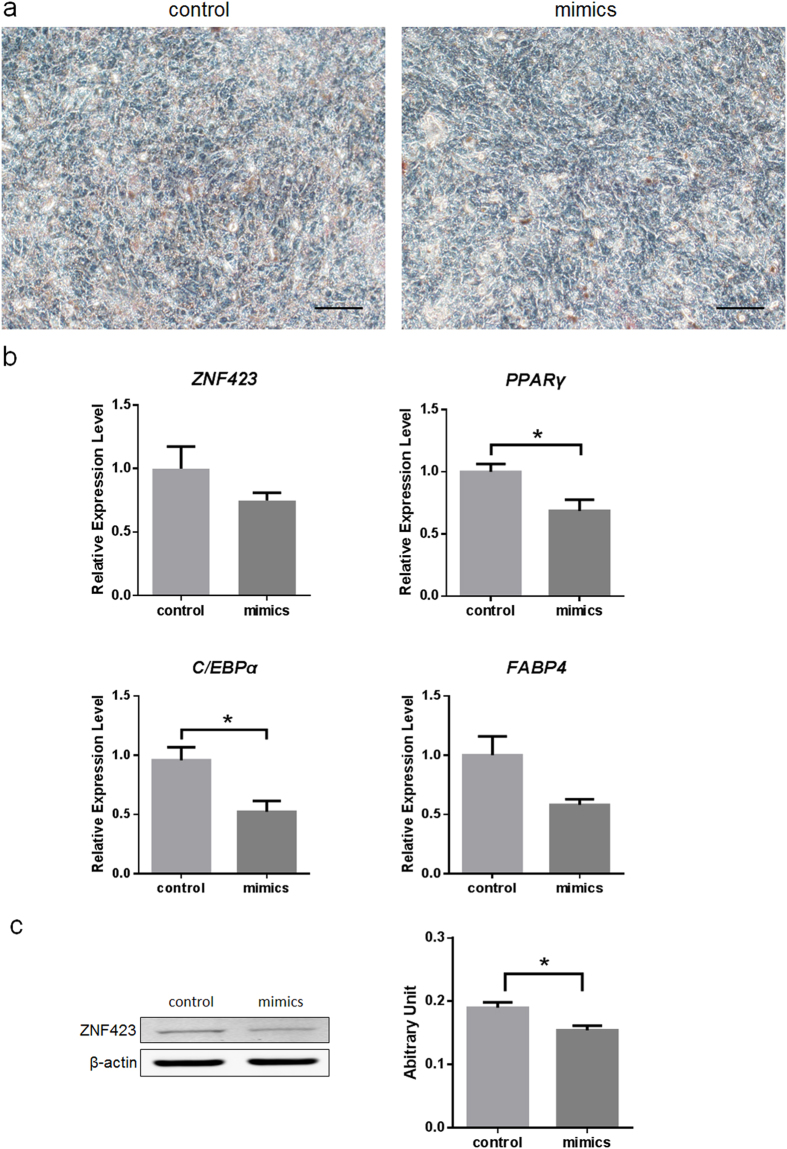
miR-23a inhibits adipogenic differentiation in PDGFRα^+^ progenitor cells. (**a**) Oil Red O staining of PDGFRα^+^ progenitor cells transfected either control or mimics at day 12. Scale bar, 100 μm. (**b**) mRNA expression of adipogenic markers at day 6. Data are presented as the means ± S.E.M. Three independent experiments are conducted in PDGFRα^+^ progenitor cells. ^*^P < 0.05. All the values were expressed relative to the control. (**c**). Immunoblot analysis of ZNF423 protein at day 12 after transfected with miR-23a mimics.

To investigate the roles of miR-23a in adipogenesis, PDGFRα^+^ progenitor cells were transfected with exogenous miR-23a mimics and induced adipogenic differentiation. Twelve days after induction, cells were conducted by Oil Red O staining. Compared with the negative control mimics group, PDGFRα^+^ progenitor cells transfected miR-23a mimics group exhibited inhibited lipid accumulation (Fig. 5a). mRNA expression of *PPARγ* and *C/EBPα* were also suppressed by the exogenous miR-23a, but *ZNF423 FABP4* expression was not different (Fig. 5b). Immunoblotting data showed that ZNF423 protein level was lower at day 12 in cells transfected miR-23a mimics (Fig. 5c).

### Inhibiting miR-23a promotes adipogenic differentiation of PDGFRα^+^ progenitor cells

**Figure 6:**
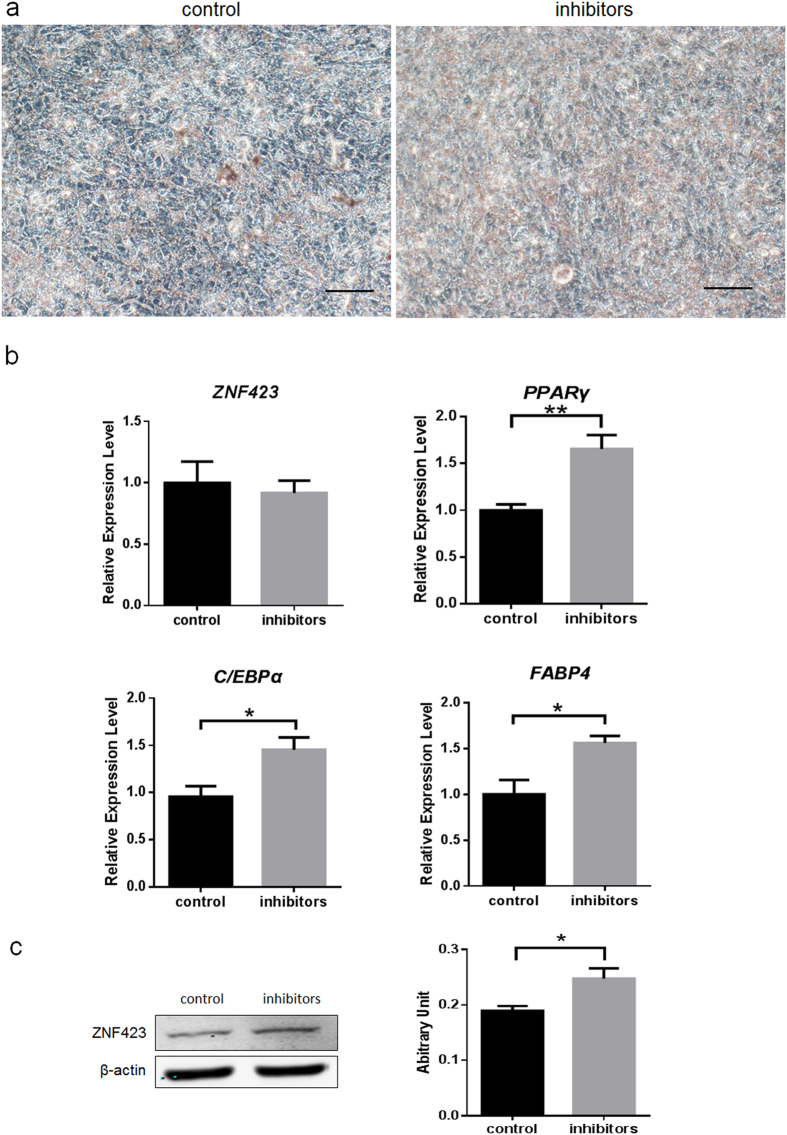
miR-23a inhibits adipogenic differentiation in PDGFRα^+^ progenitor cells. (**a**). Oil Red O staining of PDGFRα^+^ progenitor cells transfected either control or inhibitors at day 12. Scale bar, 100 μm. (**b**). mRNA expression of adipogenic markers at day 6. Expression levels were normalized using 18S. Data are presented as the means ± S.E.M. Three independent experiments are conducted in PDGFRα^+^ progenitor cells. ^*^P < 0.05, ^**^P < 0.01. All the values were expressed relative to the control. (**c**). Immunoblot analysis of ZNF423 protein at day 12 after transfected miR-23a mimics.

To further explore the function of miR-23a in adipocyte differentiation, endogenous miR-23a was knockdown by anti-miR-23a transfection at the same time of adipogenic differentiation. After 12 days differentiation, lipid accumulation was significantly increased in anti-miR-23a transfected cells compared to the control group (Fig. 6a). qRT-PCR assay showed that *ZNF423* mRNA expression did not change after miR-23a inhibitors transfection, while *C/EBPα, PPARγ* and *FABP4* expression increased significantly (p < 0.01 for *PPARγ*, p < 0.05 for *C/EBPα* and *FABP4*) (Fig. 6b). Immunoblotting assay showed that ZNF423 protein content was enhanced at day 12 after transfected miR-23a inhibitors (Fig. 6c).

### Prediction and annotation of miRNA target genes

The targets of bta-miR-23a were predicted using TargetScan. The results showed *ZNF423* was a potential target of miR-23a which could bind through a conserved site.

### miR-23a directly targets *ZNF423* in adipogenic differentiation

**Figure 7:**
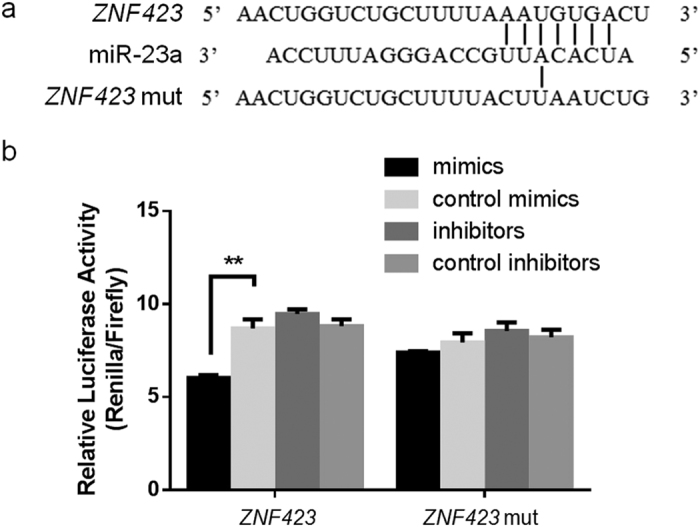
*ZNF423* is a novel target of miR-23a. (**a**) Sequences of miR-23a recognition region predicted by the Targetscan and miRanda program in the 3′ UTR of *ZNF423*. (**b**) Luciferase activities of reporter plasmids with wild type or mutated *ZNF*423 3′ UTR were examined in 293T cells by transfecting miR-23a, control mimics or control inhibitors. Data are means ± S.E.M. and were analyzed using Student’s t-test. (^**^P < 0.01).

To investigate whether *ZNF423* is a direct target of miR-23a, we constructed two luciferase reporter plasmids containing either the wild-type or mutant 3′-UTR of *ZNF423* (Fig. 7a). The luciferase reporters were co-transfected with miR-23a mimics or negative control into 293 T cell line. miR-23a significantly reduced Renilla luciferase activity of the wild-type *ZNF423* reporter compared with the negative control, while the change was not detected in the mutant luciferase reporter (Fig. 7b). These results demonstrated that miR-23a could directly target the *ZNF423* 3′-UTR.

## Discussion

Skeletal muscle is a rather complicated organ that contains various stem cell types. Satellite cell is the first identified muscle-derived stem cell and resided between the basement membrane and sarcolemma of myofiber^31^. Satellite cells stay in quiescent, but have capacity to fuse with muscle fiber when they are activated^32^, which is important for postnatal skeletal muscle growth^33^ and skeletal muscle regeneration in adulthood^34^,^35^. However, satellite cells derive from myogenic progenitor cells and do not have adipogenic potential^7^. Another type of muscle-derived stem cell with adipogenic and fibrogenic potentials is located in the stromal-vascular fraction of skeletal muscle. These adipogenic/fibrogenic progenitor cells developed from non-myogenic lineage and are capable of differentiating into IMF^7^,^36^. Although IMF is detectable as early as 180 days of gestation in bovine fetuses, adipogenic progenitor cells might differentiate from myogenic progenitor cells at an earlier time^37^. We used PDGFRα (also named CD140a) as a positive surface marker to sort the adipogenic/fibrogenic progenitor cells. PDGFRα^+^ progenitor cells were successfully induced to differentiate into adipocytes, which provided an*in vitro* model to study bovine IMF differentiation.

Next, we investigated the miRNAome expression in bovine PDGFRa^+^ cells during adipogenic differentiation. Totally, we isolated 55 miRNAs differentially expressed between un-differentiated and differentiating progenitor cells. The partial of these miRNAs have been identified as being associated with adipogenesis. miR-210 expression level was dramatically upregulated during 3T3-L1 adipogenesis^38^. miRNA-181a positively regulate adipogenesis by targeting tumor necrosis factor-α (*TNF-α*) in the porcine model^39^. During adipogenesis of human adipose tissue-derived mesenchymal stem cells, miR-221 was down-regulated^40^. In the present study, we found that bta-miR-23a was down-regulated during bovine adipogenesis. Using overexpression exogenous mimics or inhibitors of miR-23a, we demonstrated that miR-23a inhibited adipogenesis of PDGFRα^+^ progenitor cells. miR-23a is transcribed from miR-23a/27a/24–2 cluster, and processed into mature miRNAs^41^. The other two members of miR-23a/27a/24-2 cluster were also found down-regulated during adipogenesis. In addition, miR-27a has been reported regulating adipogenesis via targeting *PPARγ*^42^. miR-23a was also reported to regulate adipogenesis in 3T3-L1 cells and down-regulated during adipogenesis, but the mechanism was not clarified^43^. Here, for the first time, we report that miR-23a is a negative regulator in bovine adipogenesis by inhibiting *ZNF423* expression.

ZNF423 was identified as a key transcriptional regulator of preadipocyte determination. ZNF423 regulates *PPARγ* expression, in part, through amplification of the bone morphogenic protein (BMP) signaling pathway, an effect dependent on the SMAD-binding capacity of ZNF423^13^. Also, ZNF423 is a critical regulator of adipogenesis in stromal vascular cells (SVCs) of bovine muscle^16^. Although mechanism of transcriptional activation of *ZNF423* is not clear yet, several molecules and epigenetic modification are identified involving in the *ZNF423* regulation. ZNF521 inhibit ZNF423 directly by mediating a BMP-dependent osteoblast versus adipocyte lineage commitment switch^44^. WISP2 can prevent ZNF423 from entering nucleus by forming complex with ZNF423^45^. DNA methylation and histone methylation (H3K27me3) was also related with *ZNF423* expression^15^. So far miR-195a is the only experimentally demonstrated miRNA which targets *ZNF423*^46^. In this study, we found a new post-transcriptional regulation mechanism of *ZNF423* via microRNAs.

miR-23a has been reported with the capacity of targeting crucial proteins which regulate stem cell fate in differentiation. miR-23a is an important regulator in both osteogenesis and chondrogenesis^47^,^48^. miR-23a, which is also down-regulated during differentiation of embryonic stem cells, suppresses differentiation toward the endoderm and ectoderm lineages^49^. miR-23a also involves in the early neural differentiation by regulating Musashi1 and cyclin D1^50^,^51^ as well as erythropoiesis^52^. In this study we identified miR-23a as a regulator in early adipogenic differentiation, providing additional evidence for the biological roles of miR-23a in cell fate determination.

Because of the interaction of miR-23a and *ZNF423*, miR-23a may also potentially regulate energy metabolism and involve in the etiology metabolic diseases. In a previous study, miR-23a was isolated as a biomarker of type 2 diabetes (T2D) by comparing the serum miRNAome expression of T2D patients, pre-T2D patients and normal people^53^. This provided early diagnosis potential for the people with T2D risks. In obese individuals, a high efflux of glycerol from accumulated fat in adipose tissue into the liver is known to be associated with the development of T2D. Aquaporin 9 (AQP9) is an aquaglyceroporin family member which serves as the primary route of hepatic glycerol uptake for gluconeogenesis. miR-23a regulates glycerol-dependent gluconeogenesis by targeting *AQP9*^54^. Furthermore, miR-23a expression is down-regulated in human subcutaneous fat of obese individuals compared with lean individuals^55^. miR-23a is also differentially expressed in visceral adipose tissue between non-alcoholic steatohepatitis (NASH) and non-NASH patients^56^. Based on these findings, miR-23a is a potential biomarker of metabolism diseases. However, the biological role of miR-23a in occurrence and development of metabolism-related diseases remains to be explored.

To our knowledge, this is the first study to investigate the miRNA profiling during bovine IMF differentiation. These studies pave a new way to gain a better understanding of the regulation of miRNAs in bovine IMF development.

## Methods

### Ethics statement

Animal experiments were conducted according to the guidelines established by the Regulations for the Administration of Affairs Concerning Experimental Animals (Ministry of Science and Technology, China, 2004). All animal protocols were approved by Animal Ethics Committee of Institute of Animal Sciences, Chinese Acadamy of Agricultrual Sciences. Pregnant cows were raised in Xinrui Agriculture Co., Ltd (Weichang, China). All efforts were made to minimize the cow’s suffering.

### Progenitor cells isolation and culture

Bovine fetuses at 90 to 120 days after conception were collected immediately after removal from uterus of slaughtered cows. The fetuses were transported to the laboratory within 2–4 h. Longissimus dorsi was isolated from the fetus, minced into small fragments and then digested within Dulbecco’s modified Eagle’s medium (DMEM) containing collagenase type IV (w/v, 0.1%; C5138, Sigma, USA) for 1 h with continuous shaking at 37 °C. The digestion was neutralized by adding complete culture medium (low glucose DMEM supplemented with 10% FBS). The cell plasma was filtered through the 40 μm pore-diameter nylon meshes, collected by centrifuge and re-suspended in ice-cold phosphate-buffered saline (PBS) buffer (PBS with 0.5% bovine serum albumin and 2 mM EDTA). PDGF Receptor α antibody (#5241, Cell Signaling Technology, USA) was added to the cell suspension according to the manufacturer’s instructions and incubated at 4 °C for 30 min. The cells were washed with buffer and collected. After re-suspended in buffer, Anti-Rabbit IgG MicroBeads (#130-048-602, Miltenyi Biotec, Germany) was added and incubated for 15 min at 4 °C. Then the cells were collected and re-suspended in buffer. MACS column (#130-042-201, Miltenyi Biotec, Germany) and magnetic MiniMACS Separator (#130-042-102, Miltenyi Biotec, Germany) were used to separate PDGFRα^+^ cells. The cells were seeded in complete medium and incubated at 37 °C in a humidified atmosphere containing 5% CO_2_. At 70–80% confluence, the cells were passaged with 0.25% trypsin (0458, Amresco, USA).

### Adipogenic differentiation and Oil Red O staining

At 100% confluence, the adipogenic differentiation cocktail medium, complete medium supplemented with 10 μg/mL insulin (I5500, Sigma, USA), 1 mM dexamethasone (D1756, Sigma, USA) and 0.5 mM isobutyl-methylxanthine (I5879, Sigma, USA) was supplied to the cells while the control group was cultured in complete medium. Medium were changed every 3 days. After 12 days of differentiation, the cells were stained with Oil Red O (O0625, Sigma, USA) to assess intracellular lipid accumulation.

### RNA isolation and quality assessment

Total RNA was extracted using the TRIzol reagent (15596026, Invitrogen, USA). RNA degradation and contamination was monitored on 1% agarose gels. RNA purity was checked using the NanoPhotometer spectrophotometer (IMPLEN, CA, USA). RNA concentration was measured using Qubit RNA Assay Kit in Qubit 2.0 Flurometer (Life Technologies, USA). RNA integrity was assessed using the RNA Nano 6000 Assay Kit of the Agilent Bioanalyzer 2100 system (Agilent Technologies, USA).

### Small RNA library construction and high-throughput sequencing

A total amount of 3 μg total RNA per sample was used as input material for the small RNA library. Sequencing libraries were generated using NEBNext Multiplex Small RNA Library Prep Set for Illumina (NEB, USA.) and index codes were added to attribute sequences to each sample. Briefly, NEB 3′ SR Adaptor was directly, and specifically ligated to 3′ end of miRNA, siRNA and piRNA. After the 3′ ligation reaction, the SR RT Primer hybridized to the excess of 3′ SR Adaptor and transformed the single-stranded DNA adaptor into a double-stranded DNA molecule. 5′ends adapter was ligated to 5′ends of miRNAs, siRNA and piRNA. Then first strand cDNA was synthesized using M-MuLV Reverse Transcriptase (RNase H–). PCR amplification was performed using LongAmp Taq 2 × Master Mix, SR Primer for illumina and index (X) primer. PCR products were purified on an 8% polyacrylamide gel. DNA fragments corresponding to 140~160 bp were recovered and dissolved in 8 μL elution buffer. At last, library quality was assessed on the Agilent Bioanalyzer 2100 system using DNA High Sensitivity Chips. The clustering of the index-coded samples was performed on a cBot Cluster Generation System using TruSeq SR Cluster Kit v3-cBot-HS (Illumina) according to the manufacturer’s instructions. After cluster generation, the library preparations were sequenced on an Illumina Hiseq 2500 platform and 50 bp single-end reads were generated.

### Data processing, sRNA annotation and miRNA identification

Raw data (raw reads) of fastq format were firstly processed through custom perl and python scripts. In this step, clean datas (clean reads) were obtained by removing reads containing poly-N, with 5′ adapter contaminants, without 3′ adapter or the insert tag, containing poly A or T or G or C and low quality reads from raw data. At the same time, Q20, Q30, and GC-contents of the raw data were calculated, and a certain range of length from clean reads was set to do all the downstream analyses. The small RNA tags were mapped to reference sequence by Bowtie without mismatch to analyze their expression and distribution on the reference. Mapped small RNA tags were used to looking for known miRNA. miRBase21 was used as a reference. To remove tags originating from protein-coding genes, repeat sequences, rRNA, tRNA, snRNA, and snoRNA, small RNA tags were mapped to RepeatMasker, Rfam database or those types of data from the specified species itself.

### Differential expression analysis of miRNAs

miRNA expression levels were estimated by TPM (transcript per million) through the following criteria. Normalization formula: Normalized expression = mapped readcount/Total reads*1000000. Differential expression analysis of samples from two time points was performed using the DESeq R package v1.8.3. The P-values was adjusted using the Benjamini& Hochberg method. Corrected P-value of 0.05 was set as the threshold for significantly differential expression by default.

### Prediction of miRNA target genes

Predicting the target gene of miRNA was performed by both TargetScan^57^.

### miRNA assay using stem-loop qRT-PCR

Stem-loop RT primers and miRNA-specific primers (forward) were designed using Primer Software Primer Premier 5.0 (Premier Biosoft International, USA). U6 was used as an internal control. All the primers were listed in the Supplementary Table 2. For stem-loop qRT-PCR, approx. 1 μg of total RNA was used to create a reverse transcription pool with the TIANScript RT Kit (KR104-02, TIANGEN Biotech, Beijing). qRT-PCR was performed with the KAPA SYBR^®^ FAST qPCR Kits (KK4601, KAPA, USA), using the forward primer and a Universal Reverse Primer (Supplementrary Table 2). The cycling conditions were as follows: 3 min at 95 °C, followed by 40 cycles of 95 °C for 10 s and 60 °C for 30 s. The samples subjected to qRT-PCR were run in three biological replicates. The fold change of miRNA expression was calculated using the 2^−ΔΔCT^ algorithm^58^. The relative expression of miRNAs was normalized to the lowest expressed time point.

### Detection of mRNAs via qRT-PCR

The expression of adipogenic specific genes, peroxisome proliferator-activated receptor gamma (*PPAR-γ*), *C/EBPα* and fatty acid binding protein 4 (*FABP4*) was detected by RT-PCR. *18S* was used as an internal control. Fold change of the tested group is calculated relative to the control group using the 2^−ΔΔct^ algorithm^58^. Primers sequences are provided in Supplementary Table 2.

### Transfection of miR-23a mimics and inhibitors

miR mimic Negative Control, miR inhibitor Negative Control, mimics and inhibitors for bta-miR-23a were chemically synthesized by RiboBio Co. Ltd (Guangzhou, China). Cells were transfected using 50 nM mimics or mimics Negative Control, 100 nM inhibitors or inhibitor Negative Control according to the protocol of Lipofectamine 3000 (L3000015, Invitrogen, USA).

### DNA constructs and luciferase reporter assays

A luciferase reporter containing the wild type 3′-UTR of *ZNF423* was constructed using psi-CHECK2 vectors (Promega, USA) between XhoI and NotI restriction sites. The following site-directed mutagenesis was done using Fast Site-Directed Mutagenesis Kit (KM101, TIANGEN, China). The primers used in plasmid construction and mutagenesis are listed in Supplementary Table 2. 293T cells were co-transfected with miR-23a mimics or its inhibitors and plasmid using Lipofectamine 3000. Twenty-four hours after transfection, the luciferase activity was measured with a Dual-Luciferase Reporter Assay System (Promega, USA).

### Western Blot

Cells were lysed in lysis buffer supplemented with proteinase inhibitors. Equal amounts of total proteins were separated on 10% SDS-polyacrylamide gel. Following electrophoresis, the proteins were transferred to a polyvinylidene difluoride membrane (Millipore, USA) and blocked in 5% (w/v) non-fat milk. Membranes were incubated with antibodies against ZNF423 (sc-10486, Santa Cruz, USA), PPARγ (sc-6284, Santa Cruz, USA), FABP4 (sc-18661, Santa Cruz, USA) and β-actin (sc-47778, Santa Cruz, USA) at the concentration of 1:1000 at 4 °C overnight. The membrane was washed with Tris-buffered saline/Tween for three times and incubated with the HRP-conjugated Donkey Anti-Goat IgG (A0181, Beyotime, China) or Goat Anti-Mouse IgG (A0216, Beyotime, China) at room temperature for 1 h. The signals were visualized using ECL western blotting detection reagent (GE corporation, USA). The band intensities were quantified using Software Image Studio Lite (LI-COR Biosciences, Version 4) and protein levels were normalized to β-actin.

### Statistical analysis

Data were presented as mean ± S.E.M. Data normality was verified by Shapiro-Wilk test, followed by student’s t-test with GraphPad Prism software (GraphPad Prism, version 6.0). *P* ≤ 0.05 was considered as significant difference.

## Additional Information

**How to cite this article:** Guan, L. *et al*. bta-miR-23a involves in adipogenesis of progenitor cells derived from fetal bovine skeletal muscle. *Sci. Rep.* **7**, 43716; doi: 10.1038/srep43716 (2017).

**Publisher's note:** Springer Nature remains neutral with regard to jurisdictional claims in published maps and institutional affiliations.

## Supplementary Material

Supplementary Information

## Figures and Tables

**Table 1:** Parameters of small RNA sequences.

Samples	Reads type	Reads Number	Percentage
PC1	total reads	9232752	100.00%
	N% > 10%	59	0.00%
	low quality	26531	0.29%
	5 adapter contamine	933	0.01%
	3 adapter null or insert null	166114	1.80%
	with polyA/T/G/C	5904	0.06%
	clean reads	9033211	97.84%
PC2	total reads	8451410	100.00%
	N% > 10%	42	0.00%
	low quality	18874	0.22%
	5 adapter contamine	369	0.00%
	3 adapter null or insert null	116958	1.38%
	with polyA/T/G/C	5242	0.06%
	clean reads	8309925	98.33%
AD6d1	total reads	6409684	100.00%
	N% > 10%	73	0.00%
	low quality	15108	0.24%
	5 adapter contamine	196	0.00%
	3 adapter null or insert null	131490	2.05%
	with polyA/T/G/C	2516	0.04%
	clean reads	6260301	97.67%
AD6d2	total reads	6539308	100.00%
	N% > 10%	61	0.00%
	low quality	13115	0.20%
	5 adapter contamine	193	0.00%
	3 adapter null or insert null	333540	5.10%
	with polyA/T/G/C	2455	0.04%
	clean reads	6189944	94.66%
