# Supplementary material for: bta-miR-23a involves in adipogenesis of progenitor cells derived from fetal bovine skeletal muscle
Source: Sci Rep. 2017 Mar 3;7:43716. doi: 10.1038/srep43716 (PMC5334644; doi:10.1038/srep43716)
Supplement: Supplementary Information [file srep43716-s1.pdf]

Supplementary information

bta-miR-23a involves in adipogenesis of progenitor cells derived from fetal bovine skeletal muscle

Long Guan[1, #], Xin Hu[1, #], Li Liu[2], Yishen Xing[1], Zhengkui Zhou[1], Xingwei Liang[3], Qiyuan Yang[4], Shengyun Jin[5], Jinshan Bao[5], Huijiang Gao[1], Min Du[4], Junya Li[1, *], Lupei Zhang[1, *]

[1]Institute of Animal Science, Chinese Academy of Agricultural Sciences, Beijing 100193, China;

[2]Institute of Animal Husbandry, Heilongjiang Academy of Agricultural Sciences, Harbin 150086, China;

[3]State Key Laboratory for Conservation and Utilization of Subtropical Agro-bioresources, Guangxi High Education Laboratory for Animal Reproduction and Biotechnology, Guangxi University, Guangxi 530004, China;

[4]Department of Animal Sciences, Washington State University, Pullman, WA 99164, USA;

[5]Animal Husbandry and Veterinary Station of Wulagai, Wulagai 026321, China.

[#]These authors contributed equally to this work.

[*]Correspondence and requests for materials should be addressed to L.Z. (email: zhanglupei@caas.cn)

Supplementary Table 1. Differential expression miRNAs between PC and AD6d

miRNA	Sequence (5' to 3')	AD6d_readcount	PC_readcount	log2FoldChange	pval	padj	Regulated
bta-miR-210	ACUGUGCGUGUGACAGCGGCUGA	961.3401	22.9834	4.798	3.15E-40	8.38E-38	up
bta-miR-451	AAACCGUUACCAUUACUGAGUUU	294.7757	28.5779	3.040	1.41E-18	1.88E-16	up
bta-miR-181a	AACAUUCAACGCUGUCGGUGAGUU	15838.0904	1757.2860	2.868	1.06E-16	7.02E-15	up
bta-miR-378b	ACUUGACUUGGAGUCAGAAGGC	113.1947	6.0468	3.498	6.60E-16	3.51E-14	up
bta-miR-27a-5p	AGGGCUUAGCUGCUUGUGAGCA	258.2236	1590.5530	-2.425	5.40E-15	2.40E-13	down
bta-miR-222	AGCUACAUCUGGCUACUGGGU	1846.8565	7900.2479	-1.974	8.32E-13	3.16E-11	down
bta-miR-221	AGCUACAUUGUCUGCUGGGUUU	4601.2870	23089.2700	-2.158	1.44E-12	4.78E-11	down
bta-miR-218	UUGUGCUUGAUCUAACCAUGUG	259.4379	890.6486	-1.664	8.86E-09	2.62E-07	down
bta-miR-30a-5p	UGUAAACAUCCUCGACUGGAAGCU	10760.6547	2789.7385	1.795	1.29E-08	3.43E-07	up
bta-miR-181b	AACAUUCAUUGCUGUCGGUGGGUU	3947.4816	804.3246	2.051	1.45E-08	3.51E-07	up
bta-miR-29b	UAGCACCAUUUGAAAUCAGUGUU	192.1425	37.1852	2.068	1.28E-07	2.83E-06	up
bta-miR-184	UGGACGGAGAACUGAUAAGGGU	580.1123	3890.1042	-2.230	1.09E-06	2.22E-05	down
bta-miR-2484	GAGCUAUGAUGACUUUGAUUGCAU	95.5910	16.2280	2.125	1.28E-06	2.43E-05	up
bta-miR-17-5p	CAAAGUGCUUACAGUGCAGGUAGU	1426.1395	3913.7098	-1.366	1.55E-06	2.74E-05	down
bta-miR-101	UACAGUACUGUGAUAACUGAA	11408.2645	3591.5055	1.515	7.71E-06	1.21E-04	up
bta-miR-129-3p	AAGCCCUUACCCCAAAAAGCAU	124.4540	32.2174	1.714	7.51E-06	1.21E-04	up
bta-miR-122	UGGAGUGUGACAAUGGUGUUUG	732.1834	152.8711	1.904	8.29E-06	1.23E-04	up
bta-miR-2344	GCACGAUGAUGGCGGAUCUGAGUU	64.8159	204.0588	-1.503	8.93E-06	1.25E-04	down
bta-miR-582	UUACAGUUGUUCAACCAGUUACU	39.9004	4.8253	2.256	9.74E-06	1.30E-04	up
bta-miR-145	GUCCAGUUUUCCCAGGAAUCCCU	35358.8084	9485.1062	1.667	1.46E-05	1.85E-04	up
bta-miR-1839	AAGGUAGAUAGAACAGGUCUUGUU	1693.4533	522.3004	1.526	1.61E-05	1.94E-04	up
bta-miR-1260b	AUCCCACCACUGCCACCA	34.3896	119.5536	-1.586	2.59E-05	2.99E-04	down
bta-miR-23a	AUCACAUUGCCAGGGAUUUCCA	4365.0607	12387.0881	-1.372	3.79E-05	4.20E-04	down
bta-miR-15b	UAGCAGCACAUCAUGGUUUACA	165.4260	472.5806	-1.370	7.21E-05	7.67E-04	down
bta-miR-378	ACUGGACUUGGAGUCAGAAGGC	3275.5241	9318.5380	-1.361	9.71E-05	9.93E-04	down
bta-miR-30e-5p	UGUAAACAUCCUUGACUGGAAGCU	4124.7910	1326.0284	1.450	1.09E-04	1.07E-03	up
bta-miR-1	UGGAAUGUAAAGAAGUAUGUAU	568.7828	1410.3885	-1.200	2.41E-04	2.29E-03	down
bta-miR-132	UAACAGUCUACAGCCAUGGUCG	184.3550	31.2416	1.883	2.65E-04	2.37E-03	up

bta-miR-6119-3p	GCAAAUCAUUUUUUACUCUCCAA	31.2633	115.7787	-1.579	2.68E-04	2.37E-03	down
bta-miR-146b	UGAGAACUGAAUUCCAUAGGCUGU	68.8427	17.3355	1.630	3.16E-04	2.71E-03	up
bta-miR-92b	UAUUGCACUCGUCCCGGCCUCC	72.5048	220.9383	-1.397	4.12E-04	3.42E-03	down
bta-miR-21-5p	UAGCUUAUCAGACUGAUGUUGACU	3345085.4976	1621845.6130	0.981	4.79E-04	3.86E-03	up
bta-miR-27a-3p	UUCACAGUGGCUAAGUUCCG	1887.4262	3796.2804	-0.947	7.74E-04	6.06E-03	down
bta-miR-21-3p	AACAGCAGUCGAUGGGCUGUCU	102.0306	25.6579	1.574	9.99E-04	7.59E-03	up
bta-miR-7857	AUAGCCAGUUGGGGAAGAAUGC	214.1378	527.9305	-1.167	1.12E-03	8.26E-03	down
bta-miR-212	ACCUUGGCUCUAGACUGCUUACU	55.9568	9.5260	1.747	1.31E-03	9.41E-03	up
bta-miR-24-3p	UGGCUCAGUUCAGCAGGAACAG	10419.6909	22941.2639	-1.043	1.36E-03	9.54E-03	down
bta-miR-215	AUGACCUAUGAAUUGACAGACA	100.8955	41.7612	1.137	1.71E-03	1.17E-02	up
bta-miR-193a-5p	UGGGUCUUUGCGGGCGAGAUGA	128.9691	272.8629	-0.992	2.15E-03	1.43E-02	down
bta-let-7i	UGAGGUAGUAGUUUGUGCUGUU	299952.3595	146192.9536	0.957	2.29E-03	1.49E-02	up
bta-miR-2477	GUGGAAUGAUGAUAAGUCUGACG	10.2161	44.7881	-1.545	2.93E-03	1.82E-02	down
bta-miR-6119-5p	AGAGGUAAAAAAUUGAUUUGACU	981.4302	2301.8634	-1.094	2.95E-03	1.82E-02	down
bta-miR-2478	GUAUCCCACUUCUGACACCA	299.5252	718.2362	-1.110	3.74E-03	2.26E-02	down
bta-miR-133a	UUUGGUCCCCUUCAACCAGCUG	26.5104	72.0128	-1.217	4.01E-03	2.37E-02	down
bta-miR-664b	UAUUCAUUUAUCUCCCAGCCUAC	84.7122	25.3373	1.369	4.38E-03	2.53E-02	up
bta-miR-331-3p	GCCCCUGGGCCUAUCCUAGAA	167.7311	67.0877	1.134	5.76E-03	3.26E-02	up
bta-miR-1246	AAUGGAUUUUUGGAGCAGG	71.2588	19.4935	1.394	6.29E-03	3.49E-02	up
bta-miR-328	CUGGCCCUCUCUGCCCUUCCGU	230.0674	106.6019	0.989	6.98E-03	3.79E-02	up
bta-miR-423-5p	UGAGGGGCAGAGAGCGAGACUUU	4238.6926	2155.2406	0.891	7.22E-03	3.84E-02	up
bta-miR-125b	UCCCUGAGACCCUAACUUGUGA	7870.4248	13699.7982	-0.751	7.68E-03	3.93E-02	down
bta-miR-136	ACUCCAUUUGUUUUGAUGAUGGA	126.9653	40.2229	1.296	7.66E-03	3.93E-02	up
bta-miR-24	GUGCCUACUGAGCUGAUAUCAGU	91.4214	43.0895	0.969	7.89E-03	3.96E-02	up
bta-let-7d	AGAGGUAGUAGGUUGCAUAGUU	1503.3814	2740.6052	-0.801	9.66E-03	4.67E-02	down
bta-miR-2898	UGGUGGAGAUGCCGGGGA	781.4526	1421.8621	-0.799	9.66E-03	4.67E-02	down
bta-miR-34a	UGGCAGUGUCUUAGCUGGUUGU	1081.4353	494.4004	0.990	1.04E-02	4.93E-02	up

Supplementary Table 2. Sequences of Primers

RT primers and specify primers for miRNAs stem-loop qRT-PCR	
Name	Primer sequence(5’-3’)
U6 Forward Primer	GCTTCGGCAGCACATATACTAAAAT
U6 RT & Reverse Primer	CGCTTCACGAATTTGCGTGTCAT
miR-23a RT Primer	CTCAACTGGTGTCGTGGAGTCGGCAATTCAGTTGAGTGGAAATC
miR-23a Forward Primer	CCGAGTCAG-ATCACATTGCCAGG
miR-181a RT Primer	CTCAACTGGTGTCGTGGAGTCGGCAATTCAGTTGAGAACTCACC
miR-181a Forward Primer	CGAGTCAGAACATTCAACGCTGTC
Universal Reverse Primer	CTCAACTGGTGTCGTGGAGTCG
Primers for mRNA qPCR	
Name	Primer sequence(5’-3’)
PPARγ Forward	TGGAGACCGCCCAGGTTTGC
PPARγ Reverse	AGCTGGGAGGACTCGGGGTG
C/EBPα Forward	TGCGCAAGAGCCGGGACAAG
C/EBPα Reverse	AGCTGGGAGGACTCGGGGTG
FABP4 Forward	GGATGATAAGATGGTGCTGGA
FABP4 Reverse	ATCCCTTGGCTTATGCTCTCT
Zfp423 Forward	GGATTCCTCCGTGACAGCA
Zfp423 Reverse	TCGTCCTCATTCCTCTCCTCT
18S Forward	GTAACCCGTTGAACCCCATT
18S Reverse	CCATCCAATCGGTAGTAGCG
Primers for plasmids construction and mutagenesis	
Name	Primer sequence(5’-3’)
Zfp423-XhoI	ACTCTCGAGTAGAGAAACCCGCTTTTGGA
Zfp423-NotI	ATAGCGGCCGCCTGCAGTCAGATTAAGTCACA
Zfp423-mut Forward	GGATGGTCTGCTTTTACTTAATCTGACTGCAG
Zfp423-mut Rewerse	CTGCAGTCAGATTAAGTAAAAGCAGACCATCC

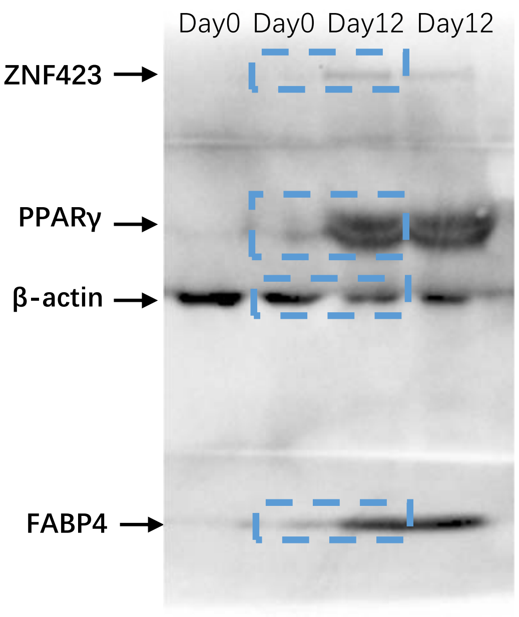

Supplementary Figure S1. Original immunoblotting data for Fig. 1. The blots of target protein were indicated with arrows. The cropped blots, displayed in the Figure 1C, were shown in blue rectangle.

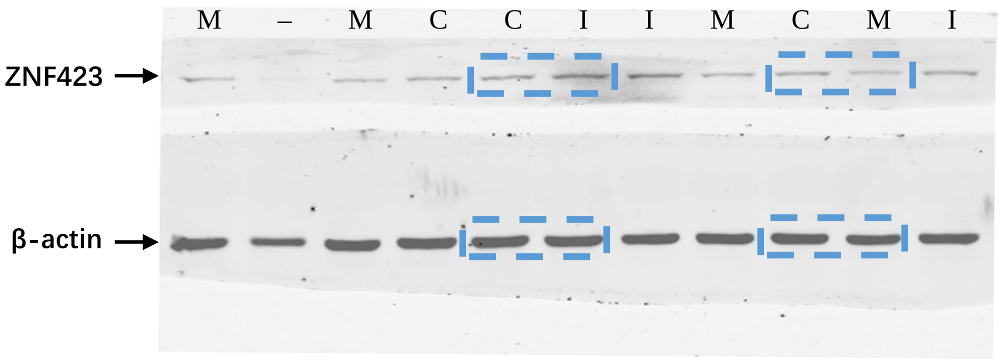

Supplementary Figure S2. Original immunoblotting data for Fig. 5 and Fig. 6. The blots of target protein ZNF423 and β-actin were indicated with arrows. The cropped blots, displayed in the Fig. 5C and Fig.6C, were shown in blue rectangle. (M, mimics; C, negative control; I, inhibitors)
